# Determining the location and nearest neighbours of aluminium in zeolites with atom probe tomography

**DOI:** 10.1038/ncomms8589

**Published:** 2015-07-02

**Authors:** Daniel E. Perea, Ilke Arslan, Jia Liu, Zoran Ristanović, Libor Kovarik, Bruce W. Arey, Johannes A. Lercher, Simon R. Bare, Bert M. Weckhuysen

**Affiliations:** 1 Pacific Northwest National Laboratory, Environmental Molecular Science Laboratory, 3335 Innovation Boulevard, Richland, Washington 99352, USA; 2 Pacific Northwest National Laboratory, Institute for Integrated Catalysis, 902 Battelle Boulevard, Richland, Washington 99352, USA; 3 Faculty of Science, Debye Institute for Nanomaterials Science, Utrecht University, Universiteitsweg 99, Utrecht 3584 CG, The Netherlands; 4 Department of Chemistry, TU Munich, Lichtenbergstrasse 4, Garching 85748, Germany; 5 UOP LLC, a Honeywell Company, 25 E. Algonquin Road Des Plaines, Illinois 60016, USA

## Abstract

Zeolite catalysis is determined by a combination of pore architecture and Brønsted acidity. As Brønsted acid sites are formed by the substitution of AlO_4_ for SiO_4_ tetrahedra, it is of utmost importance to have information on the number as well as the location and neighbouring sites of framework aluminium. Unfortunately, such detailed information has not yet been obtained, mainly due to the lack of suitable characterization methods. Here we report, using the powerful atomic-scale analysis technique known as atom probe tomography, the quantitative spatial distribution of individual aluminium atoms, including their three-dimensional extent of segregation. Using a nearest-neighbour statistical analysis, we precisely determine the short-range distribution of aluminium over the different T-sites and determine the most probable Al–Al neighbouring distance within parent and steamed ZSM-5 crystals, as well as assess the long-range redistribution of aluminium upon zeolite steaming.

## Abstract (web summary)

Substitution of framework silicon for aluminium in zeolites affects Brønsted acidity and subsequently catalytic activity. Here, the authors use atom probe tomography to obtain quantitative insights into the spatial distribution of individual aluminium atoms, including their distribution and segregation.

## Graphical abstract

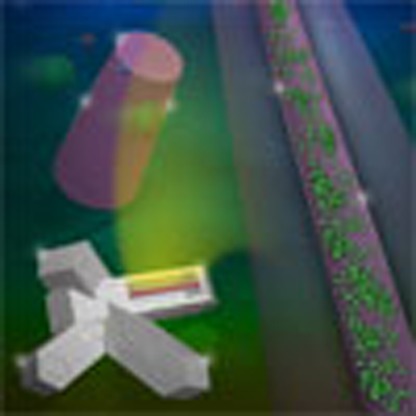

Zeolites are microporous crystalline aluminosilicates that find widespread application in the chemical and petrochemical industry, for example, as a workhorse in the catalytic cracking of heavy crude oil fractions to make gasoline and propylene. More recently, zeolite-based catalysts have found their way in the transformation of alternative feedstocks, such as biomass and natural gas. Aluminium is the key element that introduces negative charge into the framework of zeolite, which is often counterbalanced by protons leading to the formation of Brønsted acid sites that are responsible for catalytic activity. Another example of important Al-related species in zeolites include Lewis acid sites, and extra-framework metal cations that are coordinated to Al atoms in zeolites^1^. Concentration (often expressed as Si/Al ratio) and precise location of Al in the zeolite framework determine the number and strength of these acid sites^2^ and have detrimental effects on the success of various post-treatment methods that aim to improve molecular transport through the zeolite crystals, such as desilication and dealumination^3^,^4^.

The inhomogeneous distribution of Al within zeolites has been noted in numerous occasions and depends on the type of zeolite framework, Si/Al ratio, crystal size and synthesis parameters^5^ leading to distinct Al distributions that are characterized by either short-range (T-site distribution) or long-range (zoning or extra-framework) lengthscales. The unit cell of the industrially important zeolite ZSM-5 (refs 6, 7, 8) has 12 distinct crystallographic T-sites^9^,^10^,^11^. Using techniques, such as X-ray diffraction, the distribution of Al atoms over specific T-sites is difficult to determine due to low scattering contrast between Si and Al. However, X-ray diffraction studies that include ion-exchanged Cs-ZSM-5 and Tl-ZSM-5 zeolites have shown a non-random Al distribution over crystallographic T-sites^12^,^13^. Correspondingly, Al nuclear magnetic resonance (NMR) studies suggest that the Al distribution over the T-sites is kinetically controlled and not random, while the distribution can vary substantially according to the conditions of zeolite synthesis^14^,^15^. A recent study by Vjunov *et al.*^16^ confirms that identical zeolite types may have drastically different Al distribution over T-sites. Unfortunately, to date, the fundamental question as to the precise distribution of Al as a function of synthesis and how the distribution may change after processing remains elusive.

Another distinct type of Al distribution is commonly known as Al zoning and describes the long-range heterogeneous distribution of Al. Several studies have shown heterogeneous surface and bulk distribution of Al within individual zeolite ZSM-5 crystals^17^,^18^,^19^,^20^,^21^,^22^. Post-synthesis processing to either stabilize/activate or deactivate the ZSM-5 also affects the long-range Al distribution through dealumination—a process in which aluminium is expelled from the framework structure in the presence of, for example, steam, leading to the generation of extra-framework Al^23^. It is often speculated that the number and coordination of extra-framework Al species play a significant role in acid-catalysed reactions^24^,^25^,^26^.

Current characterization tools for studying the spatial distribution of Al in zeolites are scarce. Most of the methods rely on the bulk characterization of Si and Al coordination. The major drawback of bulk characterization methods is the averaging over the whole crystalline bulk, while any atomic-scale variation in the distribution of Al goes undiscovered. For example, solid-state^27^Al MAS NMR can provide detailed information about T–O–T angle and chemical nature of Al species^27^; however, the signal is averaged over the crystalline bulk and the analysis of T-site distribution still remains a difficult task. Furthermore, X-ray and electron probe-based techniques give no distinguishable contrast between Si and Al making high-resolution analysis very challenging. X-ray absorption fine structure analysis in combination with NMR was recently used to quantitatively probe bulk distribution of Al over T-sites in H-β zeolite^16^. Other X-ray absorption fine structure studies have been used to determine *in situ* structural changes of Al coordination in various zeolite topologies^28^,^29^,^30^,^31^,^32^. X-ray standing waves have been applied as a method to determine T-position of Al in extremely large crystals of zeolite scolecite^33^, while X-ray tomography in the soft X-ray regime has allowed spatial mapping of aluminium in zeolite ZSM-5, but with a limited spatial resolution of ∼30 nm (refs 34, 35). X-ray photoelectron spectroscopy^20^,^36^,^37^ and energy-dispersive X-ray spectroscopy^19^ are often used to provide surface and bulk characterization of Si/Al ratio and coordination of Al^37^ with penetrating depths of about 10 nm and 1 μm, respectively.

It is clear that the spatial placement of Al in zeolite materials is still unknown as the characterization tools that can spatially resolve the distribution of Al in zeolites are lacking. Here we present a unique characterization approach, which involves the combination of well-defined zeolite ZSM-5 crystals and a powerful nanoscale probing method, namely atom probe tomography (APT). APT is a three-dimensional (3D) compositional mapping technique based on the field evaporation, time-of-flight detection and 3D reconstruction of individual atoms (ions) from the tip of a needle-shaped specimen^38^. APT is used to quantitatively determine the 3D distribution and extent of aggregation of individual Al atoms within parent and steam-treated ZSM-5 crystals. Not only can we precisely determine the short-range distribution of Al over the different T-sites in both sets of materials and determine the most probable Al–Al neighbouring distance, but we can also assess the long-range Al redistribution upon zeolite steaming.

## Results

### FIB-based specimen preparation for APT analysis

**Figure 1:**
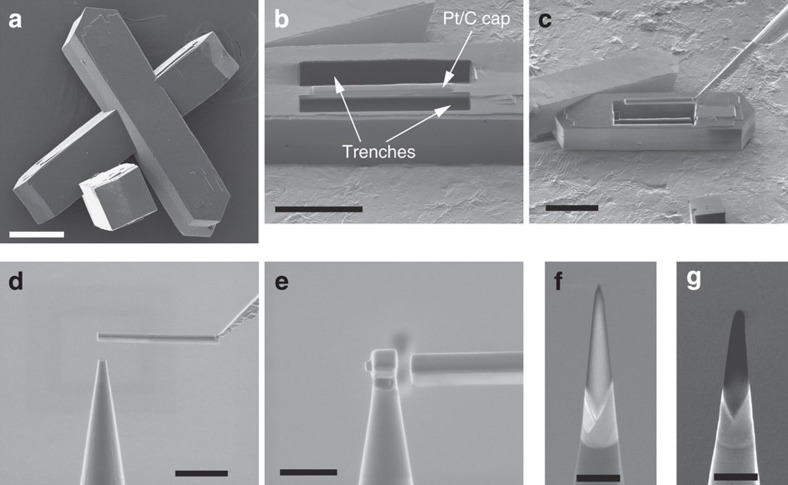
FIB-based preparation of specimens for APT analysis. (**a**) Helium ion microscope image of typical coffin-shaped zeolite ZSM-5 crystals under study. Scale bar, 20 μm. (**b**) First step in the APT specimen preparation using the FIB-SEM approach: trenching of lamellar wedge with a protective Pt cap. Scale bar, 20 μm. (**c**) Lift-out. Scale bar, 25 μm. (**d**,**e**) Attachment to Si micropost. Scale bar, 15 and 5 μm, respectively. (**f**) Final specimen morphology with tip diameter of ∼100 nm. (**g**) Same tip shown in **f** but after APT analysis. An estimate of the analysed volume is made by comparison of **f**,**g**. Scale bar for **f**,**g**), 500 nm.

For a complete description of the ZSM-5 crystal synthesis and related steam treatment used in this study, we refer the reader to ref. 36. In what follows, we will refer to ZSM-5-P and ZSM-5-ST when discussing, respectively, the parent and severely steam-treated ZSM-5 crystals under study. The typical morphology of the two sets of ZSM-5 crystals is shown in Fig. 1a, which exhibit a coffin-like geometry and ∼20 × 20 × 100 μm^3^ dimensions. We note that no distinguishable difference in the bulk morphology between both ZSM-5-P and ZSM-5-ST was observed when imaged via helium ion microscopy or scanning electron microscopy (SEM) at a nominal spatial resolution of ∼1 nm.

To prepare specimens from a portion of individual ZSM-5 crystals into the needle-like geometry necessary for APT analysis, a dual-beam focused ion beam/scanning electron microscope (FIB/SEM) was used. In brief, an electron beam and ion beam deposited a Pt/C cap of dimensions 2 × 30 μm^2^ with ∼500 nm total thickness centred longitudinally, which protects the underlying region of interest from unintentional Ga-ion damage during trenching to form a lamellar structure (Fig. 1b). An OmniProbe nanomanipulator was then attached to the lamella and it was lifted out (Fig. 1c), and ∼2–3 μm portion was positioned on a Si micropost structure (Fig. 1d,e). Using an annular milling pattern, the specimen was milled into a needle-like shape with a final tip radius curvature of ∼50 nm (Fig. 1f). Note that from a single lamella 30 μm long, ∼9–11 individual microposts can be populated and shaped into needles for analysis. Before loading the specimen substrate into the atom probe, a thin Cr film ∼20 nm thick was deposited uniformly to the surface of the specimen to increase the specimen evaporation/yield during APT analysis^39^.

Maintaining the crystallinity of the ZSM-5 sample was found to be relatively sensitive to the Ga-ion beam current. A high ion beam current of 1 nA at 30 kV during the trenching of specimen wedge (Fig. 1b) resulted in a fully amorphized structure as shown in Supplementary Fig. 1a–c. Using a relatively low ion beam current of 240 pA at 30 kV during the trenching resulted in a mostly crystalline structure as shown in Supplementary Fig. 1d,e. Interestingly, at the lower ion beam current, we consistently found an amorphous ‘shell' surrounding a crystalline core (Supplementary Fig. 1d). The thickness of the amorphous region between the Pt/C cap and crystalline core ranged from ∼40 to 100 nm, while the amorphous layer thickness on the surface ranged between ∼5 and 20 nm. With both the high and low ion beam current cases, the same 2 kV exposure was used to remove the damaged surface layer (see Thompson *et al.*^40^) and so we conclude that this low kV exposure did not result in amorphization; rather, the tendency to fully amorphize was sensitive to the ion beam current during the trenching step.

### APT of parent and severely steamed-treated ZSM-5 crystals

**Figure 2:**
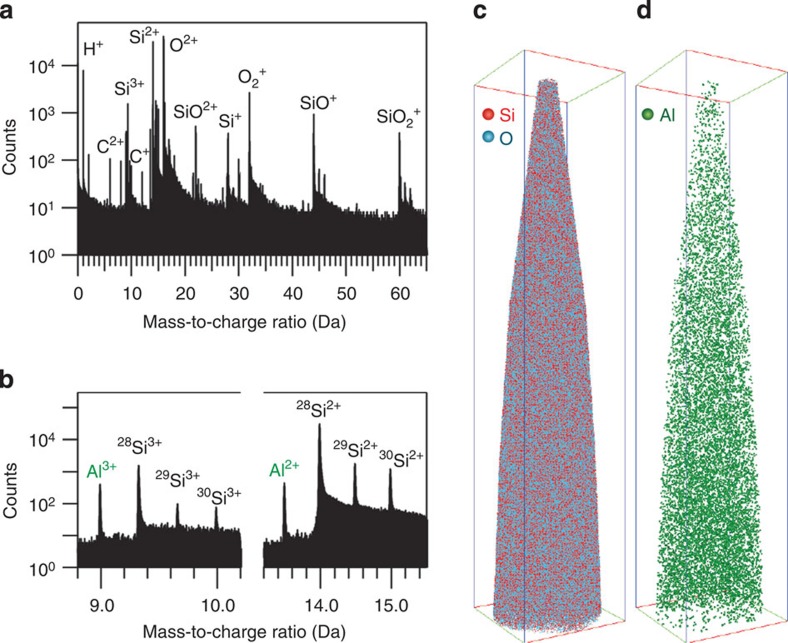
Detection and homogeneous distribution of individual aluminium atoms in parent ZSM-5-P. (**a**) Typical APT mass spectrum showing Si and O and related complex ion mass peaks. (**b**) Mass spectrum of selected region around the detected Al peaks. (**c**,**d**) 3D atom distribution map of Si and O and Al from within a parent zeolite ZSM-5 crystal. Bounding box dimensions are 60 × 59 × 298 nm^3^. Supplementary Movie 1 showing the 3D rotation of **d** is available online.

APT allows both a qualitative and quantitative analysis. Qualitatively, the distribution of Al within the ZSM-5-P crystal is found to be homogeneous as shown in Fig. 2. A mass spectrum is shown in Fig. 2a where both Si and O are observed as the major species, with Si detected as singly, doubly and triply charged ions. Complex ions made up of Si and O are also observed, for example, O_2_^+^, SiO^2+^ and SiO_2_^+^.

We also observe distinct C peaks in the mass spectrum. We can only speculate as to the origin of C as being either a remnant of the synthesis and subsequent calcination process or simply adsorbed hydrocarbons from air during sample handling. The presence of Al is shown in the zoomed in mass spectrum of Fig. 2b. Note that the mass resolution is high enough to easily distinguish the three individual isotopes of Si from monoisotopic Al. The tomographic reconstruction qualitatively shows a homogeneous distribution of Si and O (Fig. 2c) as well of Al (Fig. 2d), where each sphere represents the three-dimensional position of an individual colour-coded atom.

**Figure 3:**
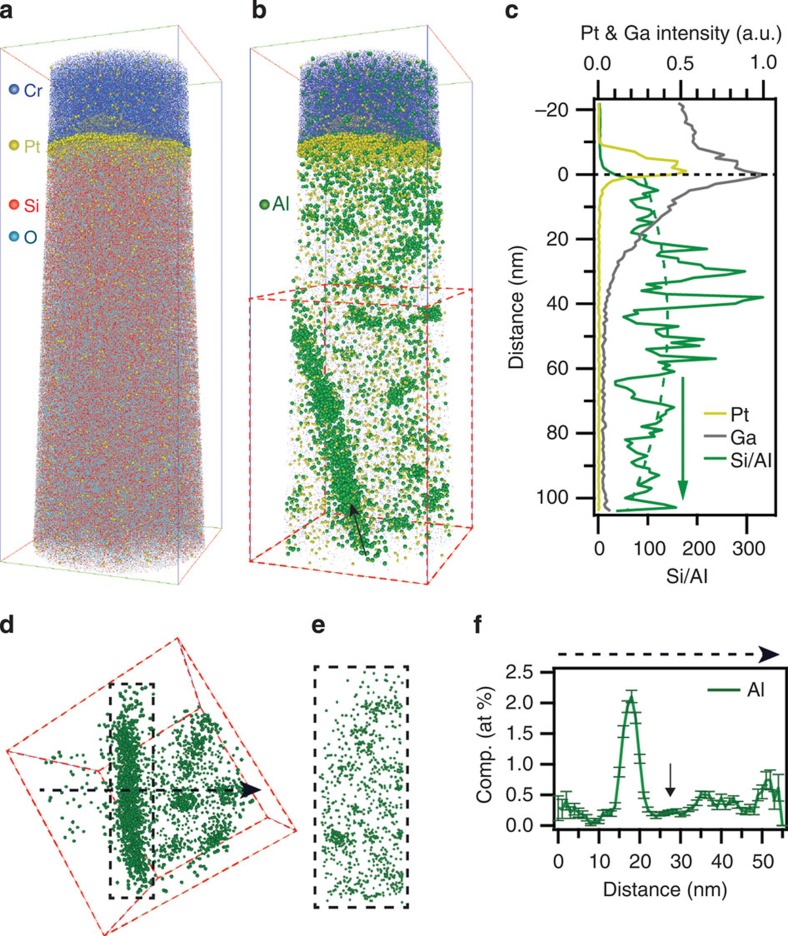
Detection and heterogeneous distribution of individual aluminium atoms in steamed ZSM-5. (**a**) Si and O and (**b**) Al atom distributions from within a steam-treated zeolite ZSM-5 crystal. The Cr and Pt layers serve as a fiducial to mark the position of the zeolite crystal surface. Bounding box dimensions are 46 × 45 × 126 nm^3^. (**c**) Si/Al ratio as a function of distance from the surface marked by black dashed line. A least-squares polynomial fit is shown as a green dotted curve to guide the eye to the distribution. The Pt and Ga intensities as well as the Si/Al ratio values are averaged over each cross-section when moving from the top to the bottom of the analysed volume. (**d**) Al distribution of isolated subvolume (red dashed box) viewed normal to the arrow direction in **b**. Bounding box dimensions 53 × 58 nm^2^. (**e**) Plane view of the isolated subvolume outlined by dashed box in **d**. Bounding box dimensions 26 × 67 nm^2^. (**f**) 1D composition profile with associated error bars taken across the arrowed black dashed line in **d**. The error bars represent the propagation point counting error as defined in the Methods section. Supplementary Movie 2 showing the 3D rotation of **b** is available online.

In stark contrast, the Al distribution in the severely steam-treated ZSM-5 crystal is found to be very heterogeneous, with regions of high Al concentration. The 3D atom maps of ZSM-5-ST in Fig. 3a,b display the distribution of Si, O and Al, respectively. Visually, the Si and O distribution is homogeneous and similar to that found in Fig. 2a for ZSM-5-P. However, the Al distribution shows a visually clear heterogeneous distribution with Al tending to form local regions of high Al concentration. In addition, we observe both the ion-sputtered Cr coating and a remnant of the FIB-deposited protective Pt capping layer that was not completely removed during the final FIB-milling. The presence of both the Cr and Pt does not negatively affect the analysis; rather, the Pt specifically provides a very important fiducial marker for quantitative compositional depth profiling relative to the initial exposed surface of the steam-treated zeolite.

In Fig. 3c, the Si/Al ratio (green) is plotted as a function of distance from the surface; the surface is marked by the horizontal dashed line below the Pt layer. Large fluctuations in the ratio are observed and arise from atomic deviations in the distribution within the 1-nm bins. Using a least-squares polynomial fit (green dashed curve), the Si/Al ratio shows a maximum of 140 at a depth of about 50 nm. We note that the unintentional implantation of Ga (Fig. 3c) during the Pt deposition has the potential to affect the Al distribution. However, it seems that any perturbation from Ga to the Si/Al ratio measured here is not significant considering our results are consistent with a previously published Si/Al ratio determined from X-ray photoemission spectroscopy depth profiling of the same steamed ZSM-5-ST sample^36^.

The individual particle-to-particle reproducibility of Al distribution in both ZSM-5-P and ZSM-5-ST as measured by APT analysis is established in the 3D reconstructions shown in Supplementary Fig. 2 for different specimens of the same ZSM-5 material. A visually homogeneous distribution of Al within the parent ZSM-5 has been observed, while Al clustering was apparent in the severely steam-treated ZSM-5, consistent with the analysis described in Fig. 2 and Fig. 3, respectively. Note that the protective Pt cap is not observed (such as the case in Fig. 3), and so the depth of the analysis relative to the exposed surface is unknown. For the ZSM-5-P data in Supplementary Fig. 2, the analysis is from a separate tip of the same individual ZSM-5-P crystal as in Fig. 2, while for the ZSM-5-ST data in Supplementary Fig. 2, the analysis is from a different individual ZSM-5-ST crystal as in Fig. 3.

As can be discerned visually in a 3D reconstruction of the steam-treated ZSM-5 sample, discrete regions of high Al concentration are found both distributed evenly in the bulk, as well as segregated along a planar feature highlighted by the black arrow in Fig. 3b. Isolation of a subvolume (Fig. 3d) reveals that the Al distribution within the planar feature occurs as discrete clusters similar to the surrounding material (Fig. 3e). In Fig. 3f, the one-dimensional (1D) composition profile taken from left to right along the red dotted line of Fig. 3d shows an approximately four times higher Al concentration within the volume of planar feature relative to the surrounding material. We suspect that this planar feature is a grain boundary between two distinct subunits that make up the monolithic coffin-shaped ZSM-5 crystals, as described by Karwacki *et al.*^20^. In fact, a slight depletion in the Al composition is observed adjacent to this planar feature (arrow in Fig. 3f), which supports Al diffusion to the grain boundary region.

**Figure 4:**
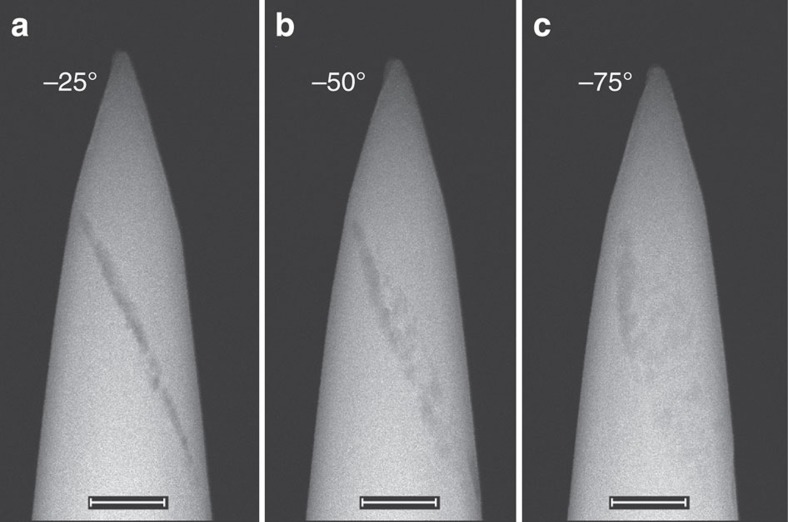
STEM characterization of a ZSM-5-ST specimen before APT analysis. (**a**–**c**) STEM images taken at the three different labelled angles using a tomography holder. Scale bar, 100 nm.

This feature is also observed through scanning transmission electron microscopy (STEM), as illustrated in Fig. 4. Figure 4a shows that at −25° the feature appears to be a line. As the specimen is rotated to −50° (Fig. 4b) and −75° (Fig. 4c), it can be seen that the line is actually made up small, finite clusters. These clusters, and their alignment in one plane, are consistent with the APT results showing the higher concentration of Al atoms in clusters that align in a plane. The darker contrast in the STEM image is indicative of voids. We postulate that these voids form along the grain boundaries during the steaming process, and the Al subsequently segregates to these locations.

### Statistical analysis of the distribution of Al in ZSM-5 crystals

Using grid-based frequency distribution analyses, the unique 3D nature of the APT data provides an unprecedented means to quantify the atomic-scale distribution of the Al atoms and the extent of segregation in the as-prepared parent and steam-treated zeolite catalysts.

**Figure 5:**
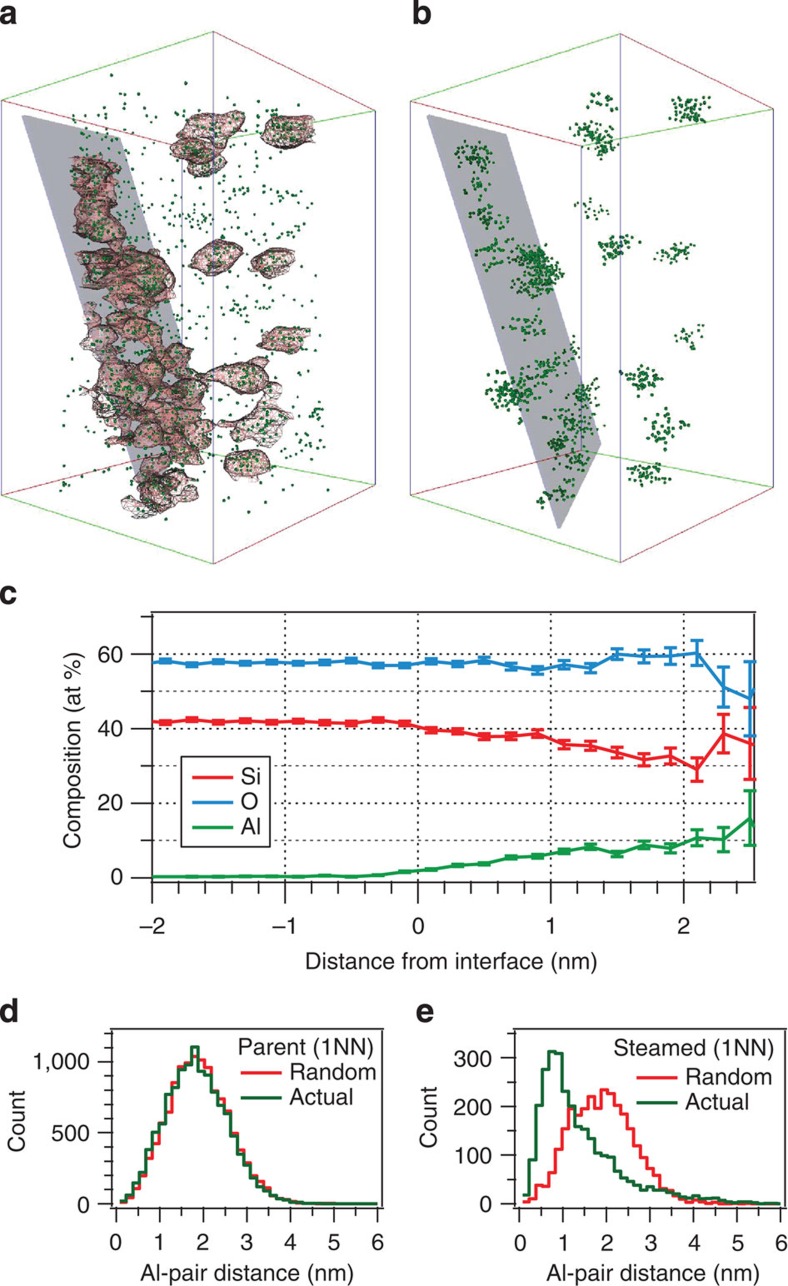
Quantification of aluminium distribution and clustering in ZSM-5-P and ZSM-5-ST. (**a**) A selected subvolume of the reconstruction shown in Fig. 3 where only isolated and clustered Al atoms (green) are shown encompassed by 2.0% Al-iso-concentration surfaces (wire mesh). A semi-transparent plane is shown to highlight the orientation of clusters lying along the grain boundary highlighted in Fig. 3b (arrow). Bounding box dimensions are 46 × 45 × 66 nm^3^. (**b**) Isolated Al solute atoms belonging to defined clusters; see text. (**c**) Proximity histogram averaged over all iso-concentration surfaces in **a**. The error bars represent 1 sigma statistical error of the averaged data set. (**d**) First Al–Al nearest-neighbour distance distributions in ZSM-5-P and (**e**) first Al–Al nearest-neighbour distance distributions in ZSM-5-ST.

An isolated subvolume of the steam-treated ZSM-5 sample is shown in Fig. 5a with 2.0% Al-iso-concentration mesh surfaces encompassing regions enriched in Al, where the volume inside (outside) is greater (less) than 2.0% Al. The heterogeneous distribution and morphology of the Al-rich regions are easily discerned. A proximity histogram (proxygram) analysis^41^ was performed for each iso-concentration surface in Fig. 5a. The analysis is similar to a 1D composition profile that follows the contours normal to iso-concentration surface. Figure 5c shows a proxygram composition profile averaged over all the surfaces shown in Fig. 5a. Over a distance of ∼1 nm, the Al composition increases to ∼10% with a correlated decrease in the Si composition. While the relative O composition remains constant, the decrease in Si with increasing Al within the Al-rich regions is expected since Al substitutes for Si in the T-sites of the ZSM-5 lattice structure.

To quantify the extent of Al enrichment, an Al–Al nearest-neighbour analysis was performed on the experimental 3D Al distribution of both parent and steamed ZSM-5 and compared with a randomized Al distribution^42^. In Fig. 5d,e, the experimental and random Al–Al first nearest-neighbour (1NN) distribution is shown for the ZSM-5-P and ZSM-5-ST material, respectively. Little deviation is visually observed between the experimental and random Al–Al 1NN distribution for the parent ZSM-5 (Fig. 5d), as opposed the steamed ZSM-5 (Fig. 5e). A *χ*^2^-statistical test was performed to quantitatively confirm or refute deviation from randomness, while a calculated Pearson coefficient (*μ*) was used to compare the relative strength of any deviation^42^. For the *χ*^2^-statistical test applied here, the null hypothesis is that the observed distribution is consistent with the expected random distribution. For the parent ZSM-5 in Fig. 5d, an experimental *χ*^2^=107 is determined, which is greater than the expected *χ*^2^=33.9 with 22 degrees of freedom and a 95% confidence level; thus, we reject the null hypothesis and confirm that the experimental Al–Al 1NN distribution is non-random.

Similarly, we quantitatively confirm a non-random Al–Al 1NN distribution for the steamed ZSM-5 in Fig. 5e from an experimental *χ*^2^=2,540 with the same expected *χ*^2^=33.9 and 22 degrees of freedom and a 95% confidence level. Although both the parent and steamed ZSM-5 Al–Al 1NN distributions show non-randomness, it is quite obvious that there is a greater departure from randomness for the steamed versus parent specimen. We can determine a relative strength of the randomness by comparing the Pearson coefficient, *μ*, whose value lies between 0 and 1, where *μ*=1 is completely non-random and *μ*=0 is completely random^43^. With a *μ*_parent_=0.7 and *μ*_steamed_=1, we confirm that there is a much stronger deviation from randomness of the steamed Al–Al 1NN experimental distribution compared with the parent distribution, which is also qualitatively apparent for the steamed specimen showing obvious regions enriched in Al.

In addition to a greater volume of regions enriched in Al, the steamed ZSM-5 also exhibits a shorter most probable Al–Al 1NN distance compared with the parent specimen. Fitting the experimental distributions in Fig. 5d,e to a Poisson distribution of the form 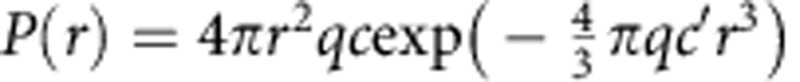 adapted from Philippe *et al.*^42^, where *q*=detector efficiency (0.36 used in the reconstructions) is fixed and *c'* is a free parameter related to the atomic density. From the relation 
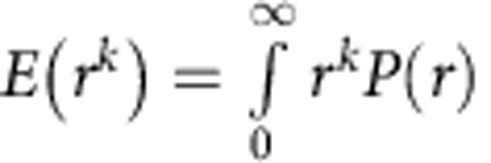
, the most probable 1NN Al–Al distance (mode) was calculated from the first moment (*k*=1) of *E*(*r*^*k*^), and s.d. (*σ*) estimated from first and second moments: 
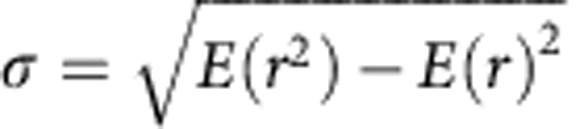
 (ref. 44). For the ZSM-5-P crystal, the mode of the Al–Al 1NN distance distribution is 18±6 Å, while for the ZSM-5-ST crystal the mode is 9±3 Å.

We note that the co-field evaporation of different phases with different evaporation fields from a heterogeneous material could lead to perturbations in local magnification resulting in the measurement of artificially diffuse distributions^45^,^46^. However, with respect to the Al–Al nearest-neighbour distance distributions reported here, we assert that local magnifications from regions of high Al content are negligible since Al does not seem to form distinct precipitates of pure Al metal or oxide.

To further quantify the Al distribution, a cluster analysis was performed following a method outlined in refs 39, 47. The cluster analysis is based on the assumption that the distance between solute (Al) atoms that are clustered together is smaller than the distance when randomly distributed in the matrix. Using a maximum spacing between two Al atoms of 1.7 nm and a minimum number of 10 Al atoms defining a cluster, 25 distinct Al clusters were found in the steamed ZSM-5 sample and are shown in Fig. 5b. We find that the Al density within the cluster is 20 times greater in the steamed sample at 0.5 atoms nm^−3^, which corresponds to 2.7 Al atoms per unit cell (UC) (with UC_ZSM-5_ taken to be *a*=20.00 Å, *b*=19.99 Å, *c*=13.40 Å), compared with 0.025 atoms nm^−3^ (0.135 Al atoms/UC) for the parent ZSM-5-P material.

## Discussion

In summary, by using the chemically sensitive method of APT for atomic-scale mapping of light elements, it is now possible to obtain unprecedented quantitative insights into the spatial distribution of individual Al atoms within zeolite crystals, including their 3D distribution and extent of segregation. It is shown that Al atoms are non-randomly distributed within the parent zeolite ZSM-5 crystals with a most probable Al–Al neighbour distance of 18±6 Å, an average Al density of 0.025 atoms nm^−3^ and a relative strength of non-randomness of 0.7 measured by the Pearson coefficient of the Al–Al 1NN distribution. In the severely steam-treated ZSM-5 crystals, the Al distribution is also non-random where Al is preferentially located in regions of relatively higher Al content of ∼10 atoms with an average Al density of 0.5 atoms nm^−3^, a most probable Al–Al neighbouring distance of 9±3 Å and a relative strength of non-randomness of 1.0. In addition, the regions of higher Al density within the steamed specimen were found to be either distributed in discrete patches within the bulk or accumulated within the grain boundaries separating the zeolite building blocks. These grain boundaries, rich in Al atoms, are proposed to be the highways for Al transport towards the outer surface of the zeolite crystals.

Our work contains important implications for zeolite-based catalysis because we observe already a non-randomness of the Al distribution within the highly crystalline parent zeolite specimen, and upon steaming this non-randomness of Al distribution further develops as a clear long-range redistribution of Al, which leads to further clustering of Al atoms. This more pronounced clustering especially occurs near regions where molecular diffusion barriers exist suggesting that these barriers, separating the zeolite subunits, act as highways for Al transport towards the outer surface of the zeolite crystals. Clearly, these observations have vast implications for industry-sized zeolite crystals as the porous network acts as highways for Al transport. Furthermore, we are convinced that the APT method offers a lot of prospect for the characterization of other zeolite-based heterogeneous catalysts currently used in chemical and oil-refining industries, making it a versatile and detailed characterization tool for assessing the processes of zeolite synthesis, steaming and deactivation.

## Methods

### Zeolite crystal synthesis and pretreatment

The coffin-shaped large ZSM-5 crystals with a Si/Al ratio of 17 were synthesized from the following raw materials: Ludox AS40, tetrapropylammonium (TPA) bromide (TPABr, Fluka), Al_2_(SO_4_)_3_·18H_2_O (Baker) and NH_4_OH (29%). The molar composition was 6.65 (NH_4_)_2_O/0.67 TPA_2_O/0.025 Al_2_O_3_/10 SiO_2_/121 H_2_O. For synthesis, Ludox AS40 and TPABr were mixed. Subsequently, Al_2_(SO_4_)_3_·18H_2_O was added and mixed for 5 min, followed by the addition of NH_4_OH and further mixing for 7 min. Heating was carried out over 2 h to 453 K (7 days soak time under static conditions), followed by washing and drying at 393 K for 12 h.

Before the steaming post-treatment, the crystals were calcined at 823 K (5 K min^−1^, 8 h) followed by triple ion exchange with a 10 wt% ammonium nitrate (**>**99%, Acros Organic) solution at 353 K. Then, the material was calcined at 773 K (2 K min^−1^, 6 h) to release the ammonia attached to the acid sites. The sample after this treatment is denoted as parent ZSM-5-P. Before starting the dealumination post-treatment, the zeolite crystals were preheated to 393 K for 60 min in a quartz tubular oven (Thermoline 79300) at a rate of 5 K min^−1^. The zeolite crystals contained inside the tubular oven were then heated to 973 K at a rate of 5 K min^−1^ and steamed using a water (373 K) saturated N_2_ flow (140 ml min^−1^) for 300 min. After the steaming post-treatment, the zeolite crystals were calcined at 773 K (5 K min^−1^, 6 h) and this sample is named steamed ZSM-5-ST.

### Zeolite crystal characterization and related analysis

The two batches of prepared zeolite crystals were imaged using a Zeiss Orion Helium Ion Microscope. An FEI Helios 600 dual-beam FIB/SEM was used to image and prepare the specimens on Si micropost arrays for APT analysis. A 30-kV Ga-ion beam was used to trench and cut the lamella and annular mill into the needle shape. A final low 2 kV shower over the tip was used to remove most of the remaining Pt/C cap and remove deep surface damage from the 30 kV beam. Microfabricated Si micropost arrays for APT analysis are commercially available from the Atom Probe Tomography division of Cameca Instruments. For a more detailed description of the FIB-based specimen preparation procedure, we refer the reader to the work of Thompson *et al.*^40^. Following FIB fabrication, a thin Cr metal film was deposited on the specimens using a South Bay Technologies IBS/e deposition system, which included simultaneous specimen rotation and tilting to ensure the conformal coating of all the needle specimens. Qualitatively, we found that APT analysis yield increased with ∼10–30 nm Cr coating. A LEAP × 4000 HR local electrode APT system from CAMECA Instruments was used to map the atomic composition of individual zeolite specimens. The following parameters were used for both ZSM-5-P and ZSM-5-ST—laser energy=200 pJ, detection rate=0.2% and temperature=44 K.

The tomographic reconstructions and analysis, including nearest-neighbour and cluster analyses, were performed with an *x*=1.0 nm (3.0 nm), *y*=1.0 nm (3.0 nm), *z*=1.0 nm (1.5 nm) voxel grid size (delocalization) using Cameca's Integrated Visualization & Analysis Software (IVAS) version 3.6.6. IVAS was also used for the cluster analysis with parameters *D*_max_=1.7 nm and *N*_min_=10. For the nearest-neighbour analysis, the expected nearest-neighbour distribution (random) was determined from a simulated random distribution of the same solute composition and reconstructed dimensions. The error bars for the 1D composition profiles reported throughout represent the propagation point counting error as 
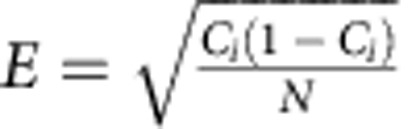
 where *C*_*i*_=(*x*_*i*_*/N*), *x*_*i*_ is the number of *i* solute ions and *N* is the total number of counts within the given bin. The error in the 1NN distances is reported as 1 s.d. The most probable distance, mode, of the Al–Al 1NN distribution was determined from distance corresponding to the value of greatest probability. When possible, a comparison of both before and post SEM images were used to estimate the volume of evaporated material and guide the choice of reconstruction parameters to obtain a reconstruction of reasonable volume (see Fig. 1f,g and Supplementary Fig. 3). For a more detailed description of atom probe analysis and data reconstruction and analysis using a LEAP, please refer to the published user's guide^39^.

## Additional information

**How to cite this article**: Perea, D. E. *et al.* Determining the location and nearest neighbours of aluminium in zeolites with atom probe tomography. *Nat. Commun.* 6:7589 doi: 10.1038/ncomms8589 (2015).

## Supplementary Material

Supplementary Figures Supplementary Figures 1-3.

Supplementary Movie 1 Animated GIF movie of Parent ZSM-5. Distribution of Al atoms (green) from Fig. 2d in the main text.

Supplementary Movie 2 Animated GIF movie of Steam Treated ZSM-5. Distribution of Al (green), Pt (yellow), Cr (blue) from Fig. 3b in the main text.
