# Supplementary material for: Determining the location and nearest neighbours of aluminium in zeolites with atom probe tomography
Source: Nat Commun. 2015 Jul 2;6:7589. doi: 10.1038/ncomms8589 (PMC4506508; doi:10.1038/ncomms8589)
Supplement: Supplementary Figures — 1-3. [file ncomms8589-s1.pdf]

Supplementary Figures

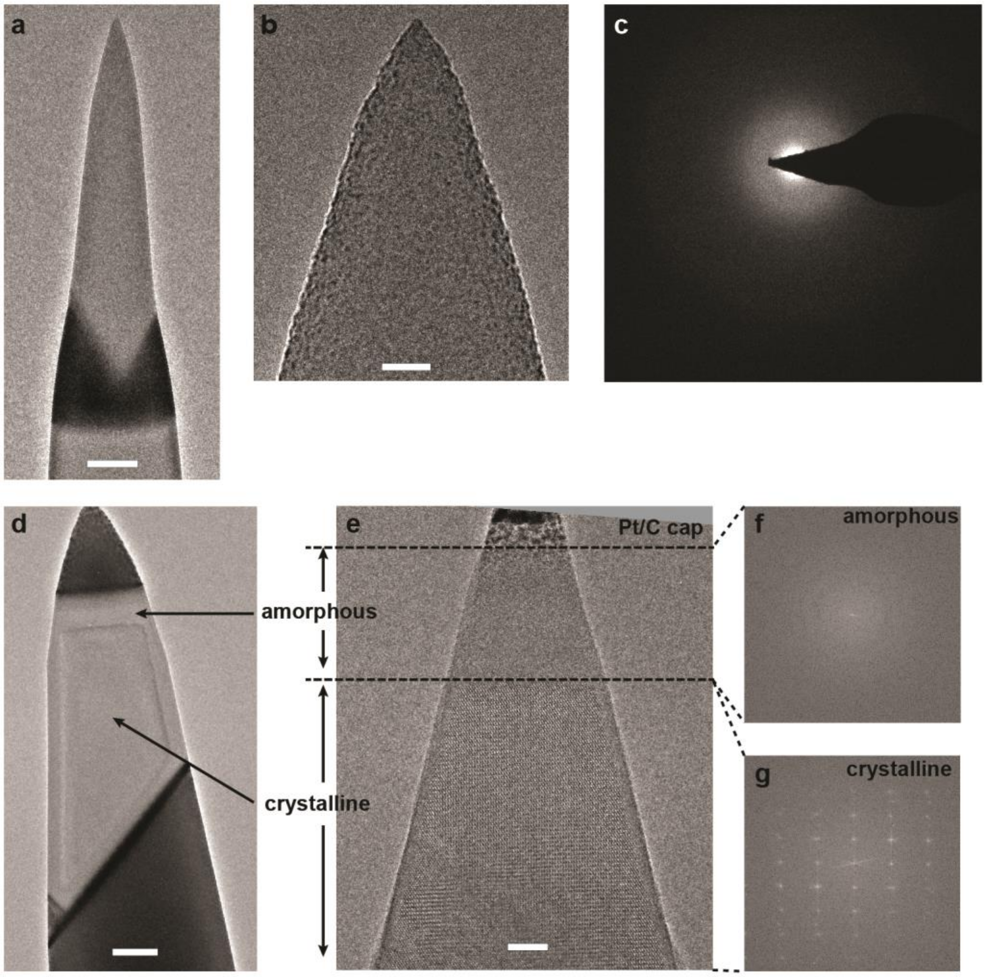

Supplementary Figure 1 | Characterization of Ga ion beam damage during FIB specimen preparation. (**a-c**) Transmission electron micrographs and related diffraction pattern from a FIB-prepared APT needle specimen using high Ga ion beam current. Scale bar in (a), 200 nm. Scale bar in (b), 20 nm. (**d-e**) Transmission electron micrographs from FIB-prepared APT needle specimens using low Ga ion beam current. Scale bar in (d), 100 nm. Scale bar in (e), 20 nm. (f) and (g) Related diffraction patterns taken from the amorphous and crystalline regions, respectively.

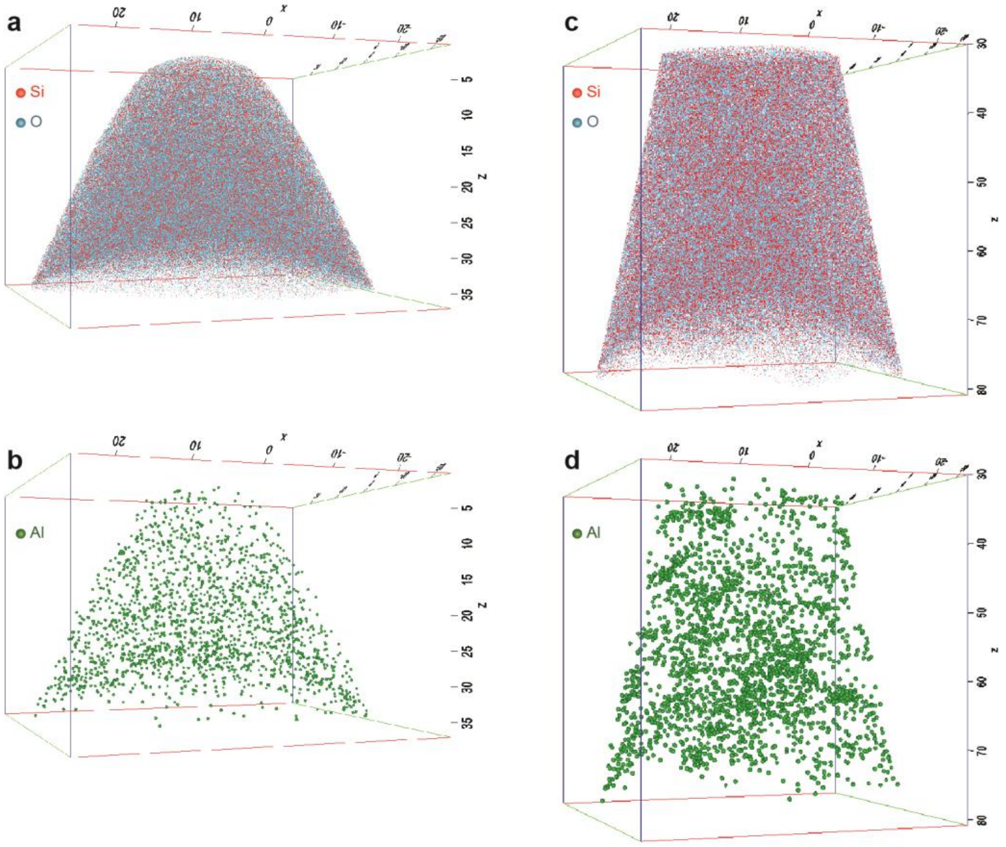

Supplementary Figure 2 | APT analysis of different specimen tips to illustrate the repeatability of the experiments performed. (**a**) Si and O, and (**b**) Al distribution in the parent ZSM-5-P material. (**c**) Si and O, and (**d**) Al distribution in the severely steam-treated ZSM-5-ST material.

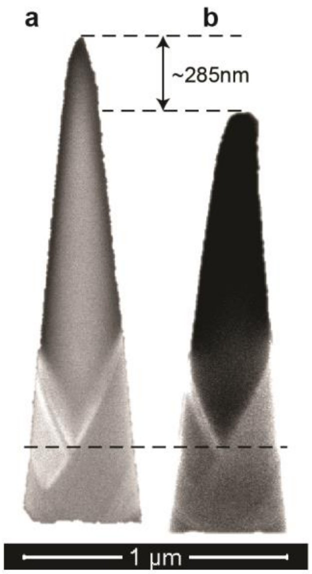

Supplementary Figure 3 | Correlative microscopy for quantitative 3-D scaling of APT data. Scanning electron microscopy (SEM) images taken before, (a), and after, (b), of the parent ZSM-5-P specimen analyzed in Fig. 2 in the main text. A quantitative estimate of the volume of evaporated material is made by comparison of the images and is used to inform the z-scaling of the reconstruction.
